# Opportunities and Challenges in Global Quantification of RNA-Protein Interaction *via* UV Cross-Linking

**DOI:** 10.3389/fmolb.2021.669939

**Published:** 2021-05-13

**Authors:** Carlos H. Vieira-Vieira, Matthias Selbach

**Affiliations:** ^1^Proteome Dynamics, Max Delbrück Center for Molecular Medicine in the Helmholtz Association, Berlin, Germany; ^2^Faculty of Life Sciences, Humboldt Universität zu Berlin, Berlin, Germany

**Keywords:** cell signaling, posttranscriptional regulation, post-translational modifications, RBPs, RNA binding proteins, RNA interactome capture, Clip, RNA-binding quantification

## Abstract

RNA-binding proteins (RBPs) are key mediators of posttranscriptional gene expression control. However, the links between cell signaling on the one hand and RBP function on the other are understudied. While thousands of posttranslational modification (PTM) sites on RBPs have been identified, their functional roles are only poorly characterized. RNA-interactome capture (RIC) and cross-linking and immunoprecipitation (CLIP) are attractive methods that provide information about RBP-RNA interactions on a genome-wide scale. Both approaches rely on the *in situ* UV cross-linking of RBPs and RNAs, biochemical enrichment and analysis by RNA-sequencing (CLIP) or mass spectrometry (RIC). In principle, RIC- and CLIP-like methods could be used to globally quantify RBP-RNA interactions in response to perturbations. However, several biases have to be taken into account to avoid misinterpretation of the results obtained. Here, we focus on RIC-like methods and discuss four key aspects relevant for quantitative interpretation: (1) the RNA isolation efficiency, (2) the inefficient and highly variable UV cross-linking, (3) the baseline RNA occupancy of RBPs, and (4) indirect factors affecting RBP-RNA interaction. We highlight these points by presenting selected examples of PTMs that might induce differential quantification in RIC-like experiments without necessarily affecting RNA-binding. We conclude that quantifying RBP-RNA interactions via RIC or CLIP-like methods should not be regarded as an end in itself but rather as starting points for deeper analysis.

## Introduction

Posttranscriptional regulation is an essential part of gene expression control (Buccitelli and Selbach, 2020), and RNA-binding proteins (RBPs) are particularly important players (Gehring et al., 2017; Gebauer et al., 2020). However, in contrast to transcription factors, the links between cell signaling events and RBP function are not well characterized. Posttranslational modifications (PTMs) of RBPs are expected to play a key role in this process. On the one hand, PTMs are key mediators of cell signaling. On the other hand, PTMs can affect the activity of RBPs (Yu, 2011; Thapar, 2015; Lovci et al., 2016). For example, PTMs have been shown to regulate RBPs in diverse cellular contexts, including protein translation (Imami et al., 2018; Jia et al., 2021), RNA stability and processing (Durand et al., 2016; Zhang et al., 2018), splicing (Stamm, 2008), and phase separation (Hofweber and Dormann, 2019).

Tens of thousands of PTM sites have been identified in the proteome, but the functional significance of the vast majority of them is currently unknown (Sharma et al., 2014; Larsen et al., 2016; Ochoa et al., 2020). A key challenge is that experimental techniques to assess the function of individual PTM sites are typically not scalable. Hence, systematic approaches to identify functionally relevant PTM sites in proteins is a topic of intense research (Imami et al., 2018; Huang et al., 2019; Masuda et al., 2020; Ochoa et al., 2020).

Since the defining feature of RBPs is their ability to bind RNA, an attractive approach to assess the function of PTM sites in RBPs would be to quantify how they affect RNA binding. Methods that employ UV cross-linking of RBPs and RNA *in situ* followed by “omic” analyses enable identification and quantification of hundreds of RBPs or thousands of RNA-binding sites in a single experiment (Wheeler et al., 2018; Lin and Miles, 2019; Gebauer et al., 2020). These experiments come in two flavors: In protein-centric methods like cross-linking and immunoprecipitation (CLIP), RBPs are purified and RNA targets identified by sequencing (Wheeler et al., 2018; Lin and Miles, 2019). CLIP-like methods provide a detailed picture of the RBP-RNA interactome with single nucleotide resolution for a specific RBP of interest (Hafner et al., 2010; König et al., 2010). Conversely, RNA-centric methods like RNA-interactome capture (RIC) use mass spectrometry-based proteomics to identify the RNA-bound proteome following biochemical isolation of RNAs (Gebauer et al., 2020; Gräwe et al., 2020). In analogy to CLIP, amino acid resolution of RBP-RNA interactions can be obtained (Kramer et al., 2014; Bae et al., 2020). Finally, related methods take advantage of specific biochemical properties of ribonucleoprotein complexes to purify both RBPs and RNAs at the same time (Smith et al., 2020). All of these methods can be categorized as CLIP- or RIC-like depending on the readout (transcriptomics or proteomics, respectively). For a detailed methodological discussion, we refer the interested reader to excellent reviews on the available methods and their limitations (Ramanathan et al., 2019; Gebauer et al., 2020; Gräwe et al., 2020; Smith et al., 2020).

Studying the impact of PTMs on RBP-RNA interactions is conceptually simple: the biological system under study is perturbed, and changes in RBP-RNA binding are studied via CLIP- or RIC-like assays. RIC-like methods are particularly attractive because the readout via mass spectrometry can be used to directly assess PTMs. However, despite this conceptual simplicity, interpreting results from such experiments can be challenging. Here, we discuss the biases involved, with the goal to highlight both the challenges and also the opportunities for systematically identifying functional PTMs in RBPs. First, we will emphasize the differences between the read-outs of CLIP- and RIC-like assays. We will then discuss specific biases that are particularly relevant for RIC-like experiments. Finally, we outline how PTMs can affect specific aspects of RBP function via known examples.

### CLIP- and RIC-Like Methods Provide Different Types of RBP-RNA Interaction Data

**FIGURE 1:**
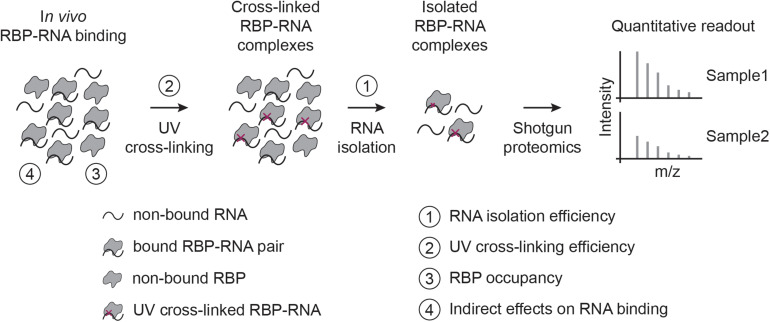
RIC-like assays and biases involved in quantifying RBP-RNA interactions. In RIC-like experiments, *in vivo* RBP-RNA interactions are stabilized by UV cross-linking. RNAs are isolated and the bound proteome is quantified with shotgun proteomics. We identify here four biases in the interpretation of RBP-RNA quantification results: RNA isolation (1), UV cross-linking (2), RBP occupancy (3), and indirect effects on RNA binding (4).

Before discussing specific biases, it is important to remember that CLIP- and RIC-like methods provide fundamentally different types of RBP-RNA measures: CLIP maps RBP binding sites globally, while RIC captures the proteins that bind to RNA. Quantitative interpretation of CLIP-like experiments is difficult (Ramanathan et al., 2019), and only performed in exceptional cases (Schueler et al., 2014) or indirectly (Gregersen et al., 2014; Milek et al., 2017; Smith et al., 2021). In contrast, RIC-like experiments are often used to assess changes in RNA-binding for RBPs across conditions (Hentze et al., 2018), and several groups have identified context-specific regulatory RBPs in mammalian tissue culture cells (Boucas et al., 2015; Liepelt et al., 2016; Milek et al., 2017; Perez-Perri et al., 2018; Garcia-Moreno et al., 2019; Ignarski et al., 2019; Queiroz et al., 2019; Trendel et al., 2019; Hiller et al., 2020; Smith et al., 2021), zebrafish and fly embryos (Sysoev et al., 2016; Despic et al., 2017), yeast (Shchepachev et al., 2019; Bresson et al., 2020), and plant cells (Marondedze et al., 2019). While it is tempting to interpret differences in RIC-like experiments as changes in RBP-RNA interaction (RNA-bound protein fraction), this is not to be taken for granted. In this perspective, we focus on UV-crosslinking-based RIC-like experiments (Figure 1), although some points raised are also relevant for CLIP-like assays and other cross-linking approaches.

### Isolation Efficiency of Bound RNAs Biases Quantification of RBP Binding

In CLIP-like assays, the pull-down efficiency of RBPs (usually with antibodies) is generally assumed to be independent of the bound RNA sequences. In RIC-like assays on the other hand, the pull-down efficiency of RBPs strongly depends on the isolation efficiency of their RNA targets (Gräwe et al., 2020). Features such as RNA length, subcellular localization, base composition, modifications and secondary structures can all influence RNA isolation. For example, RIC-like experiments using oligo(dT)-beads first isolate poly-A mRNAs through the A-T hybridization with beads (Baltz et al., 2012; Castello et al., 2012; Perez-Perri et al., 2018). In this case, the RNA isolation is affected by the A-T hybridization strength, such that there is a bias against mRNAs with shorter poly-A tails. Since the oligo(dT)-beads used for isolation are typically as short as 18–20 bases, this bias is probably mostly relevant for mRNAs with very short poly-A tails (shorter than 20 nts) (Park et al., 2016). Importantly, poly-A tail length is itself regulated during specific biological processes like the cell cycle (Park et al., 2016) and maternal to zygotic transition (Despic et al., 2017). Hence, isolation efficiency deserves special attention when analyzing such biological processes.

Oligo(dT)-enrichment is not the only RNA isolation method prone to biases (Perez-Perri et al., 2018, 2021; Scholes and Lewis, 2020). For instance, enrichment of the RBP-RNA complex using organic phase separation isolates complexes bound to RNAs as small as 30 nucleotides, but isolation efficiency drops dramatically for smaller RNAs (Urdaneta et al., 2019). Also, methods that enrich specific RNAs via hybridization to complementary oligonucleotide probes are sensitive to modifications of the RNA sequence that might impair hybridization (Gräwe et al., 2020).

### UV Cross-Linking Efficiency Is a Major Factor for RBP-RNA Quantification

RIC-like experiments rely on the ability of the bound RBP to cross-link to the RNA it is interacting with. UV cross-linking is an attractive method to study RBP-RNA interactions, mainly due to its ability to stabilize interactions *in situ* in otherwise unmodified cells or tissues (Meisenheimer and Koch, 1997; Ramanathan et al., 2019). However, multiple factors influence cross-linking efficiencies, and not all RBP-RNA pairs are cross-linked equally well.

Upon single-photon excitation with UV light (∼254 nm), atoms of the nucleotide are excited to a higher energy state for a short time period. Only during this short time period (microseconds) nucleotides can form covalent cross-links with amino acid residues in close proximity (“zero-distance”) (Budowsky et al., 1986; Meisenheimer and Koch, 1997). This is crucial for achieving high specificity but also makes the cross-linking reaction very inefficient. RBP-RNA cross-linking efficiency with continuous wave UV irradiation has been estimated to range from <0.1 to 5% (Budowsky et al., 1986; Fecko et al., 2007; Darnell, 2010). In addition to the overall low efficiency, differences exist between different RBP-RNA pairs. For example, uridines are favored *in vitro* (Meisenheimer and Koch, 1997) and are possibly the only detectable cross-linking nucleotide *in vivo* (Kramer et al., 2014; Bae et al., 2020). Also, double stranded RNAs poorly cross-link to bound proteins, and the direction with which the nucleotide makes contact (base, sugar, and phosphate backbone) also affects cross-linking efficiency (Meisenheimer and Koch, 1997). Finally, cross-linking efficiency also varies depending on the amino acid side chains in the RBP (Meisenheimer and Koch, 1997). While all amino acids have been shown to cross-link to some extent, amino acid-specific differences in cross-linking efficiency appear to exist *in vivo* (Kramer et al., 2014; Bae et al., 2020).

In summary, cross-linking efficiencies are generally low and affected by site-specific factors. The extremely low efficiency implies that minor differences in UV cross-linking can have a large impact on quantification. This point is mostly relevant when different sites are compared with each other in CLIP-like experiments. When comparing the same sites across different samples a low crosslinking efficiency *per se* is unlikely to lead to biases since it is expected to affect all samples equally.

### Baseline RBP Occupancy by RNAs Limits the Outcome in Relative Quantification

To form novel interactions with RNA in response to perturbations, RBPs must be free (that is, not RNA-bound). Therefore, the baseline occupancy of an RBP (that is, the fraction of all RBP molecules that are already bound to RNA) restricts the changes that can be observed in relative quantitative analysis: Low RBP occupancy at baseline allows larger increases, while RBPs with high baseline occupancy are already close to maximal binding, and the opposite is true for decreases in RNA-binding. It is important to consider the baseline global RBP occupancy when studying changes across conditions, as this will affect the biological interpretation of results obtained in RIC-like experiments.

**FIGURE 2:**
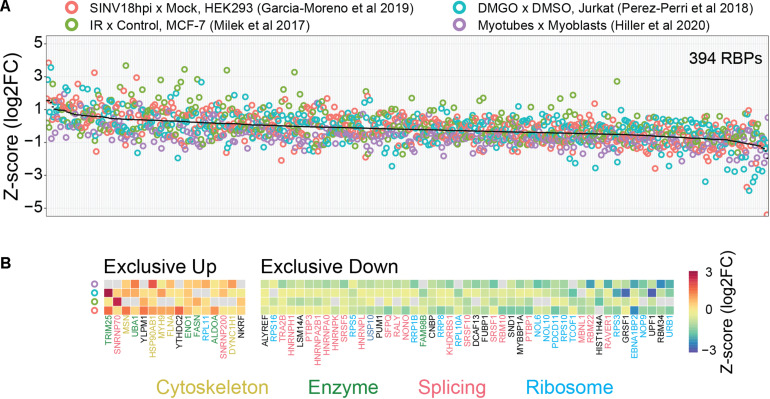
Possible link between baseline RBP occupancy and observed changes in comparative RIC experiments. We used published data from four different cellular systems and perturbations [HEK293 cells infected with Sindbis virus (SINV), Infrared (IR) radiated MCF-7 cells, DMGO treated Jurkat cells, and differentiated myoblasts (myotubes)]. **(A)** Intra-experimentally *z*-scored log2 fold changes for 394 RBPs quantified in at least three experiments. Proteins were ranked by their mean fold change (black line). **(B)** RBPs exclusively up- or down-regulated in all experiments. Protein function was annotated manually. “Cytoskeleton” includes cytoskeleton dynamics-related proteins (yellow), “Enzymes” includes metabolic enzymes and protein modifiers (green), “Splicing” includes spliceosome components and splicing-related RBPs (red), “Ribosome” includes both core ribosome components and ribosome biogenesis-related factors (light-blue). We note that “moonlighting” RBPs (Cytoskeleton and Enzymes) and “core” RBPs (Splicing and Ribosome) tend to be up- and down-regulated, respectively. See text for more details.

Consistent with the considerations above, we observed that several classical core RBPs (splicing factors, ribosomal proteins, and hnRNPs) show decreased binding in four comparative RIC studies employing different perturbations in distinct mammalian cell lines (Figure 2; Milek et al., 2017; Perez-Perri et al., 2018; Garcia-Moreno et al., 2019; Hiller et al., 2020). Conversely, proteins with moonlighting RNA-binding activity such as metabolic enzymes tend to show increased binding. It is tempting to refer to the first (core RBPs) and second (moonlighting RBPs) group of proteins as high and low baseline occupancy RBPs, respectively. These data thus support our considerations on the relationship between baseline occupancy and quantitative outcome. However, we do not know if this observation can be extended to other comparative RIC studies. It is also important to keep in mind that the situation *in vivo* is probably more complex. Most importantly, the actual baseline occupancy of RBPs is not known and also depends on the cellular context. In general, the occupancy depends on the binding affinity, the (local) concentration of the RBP and the number of available binding sites. For example, low concentration RBPs with a high number of RNA-binding sites are likely to be highly occupied. While the RNA-binding sites of a given RBP can be identified using CLIP-like methods, competition over binding sites between components in the cellular RNA network (other RBPs, RNA-RNA interactions, etc.) complicates estimating actual number of available sites for RBP interactions (Jens and Rajewsky, 2015). Measuring protein concentrations is also difficult, particularly because RBPs tend to localize in specific subcellular compartments where they exert their functions (Sundararaman et al., 2016). Finally, it is not yet possible to measure the *in vivo* RBP-RNA binding affinities. In combination, these factors complicate estimation of baseline occupancies and how this might impact the outcome of RIC-like experiments.

### Indirect Effects on RNA Binding Independent of RBP Regulation

While binding to RNA is the defining feature of all RBPs, important aspects of their cellular function (like protein–protein interaction) do not depend on changes in their interaction with RNA. Conversely, changes in RNA-binding might occur as a secondary effect of other cellular events. For example, cells typically shut down translation in response to stress, which releases mRNAs that would otherwise be bound by ribosomes (Liu and Qian, 2014; Advani and Ivanov, 2019). Cellular stress also leads to formation of stress granules, where multiple RNAs are sequestered away (Ivanov et al., 2019). In both cases, corresponding RBPs can experience drastic changes in the amount of available RNA-binding sites. The fact that RBP-RNA binding depends on the availability of RNA-binding sites also hinders comparison across conditions where transcriptomes vary greatly. This might be the case when comparing stages during embryonic development (Sysoev et al., 2016; Despic et al., 2017), viral infection (Garcia-Moreno et al., 2019), or strong cellular perturbations like arsenite-induced stress (Trendel et al., 2019). Hence, some regulatory events affecting RBP function will not be captured by quantifying changes in their RNA-binding.

### RBP Functional Regulation by PTMs

Despite the challenges outlined above, both RIC and CLIP are powerful methods that can provide information about RBP-RNA interactions on a genome-wide scale. A particularly attractive application of RIC-like methods (specially comparative RIC) is to study how RBP function is modulated by PTMs. Since most PTMs have not yet been studied via RIC-like experiments, we instead focus on exemplary cases of RBPs whose regulation by PTMs is sufficiently well characterized to allow us to speculate on their impact, taking the aforementioned biases into account.

#### RNA Affinity

The most direct way by which PTMs could affect RBP-RNA interaction is to change binding affinities. Amino acids in RBPs that directly contact RNAs are enriched in serine, threonine and tyrosine phosphorylation, lysine acetylation and arginine methylation sites (Bae et al., 2020). Several PTM sites have been shown to change the RBP affinity toward RNA targets, although that is not always the case (Thapar, 2015).

Intuitively, higher binding affinities result in increased RNA-binding (and vice-versa). However, due to the biases described above, an increased abundance in RIC-like experiments might not necessarily follow. For example, if the occupancy of the RBP is already close to maximum at baseline, major changes are not expected. Also, changes in the group of transcripts targeted by the RBP might lead to unexpected results in case this group of transcripts shows different isolation and/or UV cross-linking efficiencies. LARP1 might be a good example for the latter case: Upon inhibition of the upstream kinase mTORC1, the abundance of LARP1 in RIC-like experiments increases (Smith et al., 2020), suggesting stronger RNA-binding. While LARP1 interacts in cells with multiple transcripts (Hong et al., 2017), mTORC1-dependent phosphorylation modulates affinity toward the specific group containing the 5′ TOP motif (Jia et al., 2021). Whether 5′ TOP motif RNAs have different isolation/cross-linking efficiency is not known. Interestingly, both phosphorylation sites that increase or decrease affinity toward TOP mRNAs are regulated by mTORC1. Instead of increasing the mRNA-bound protein fraction as suggested by the higher abundance in RIC-like experimental results, mTORC1-induced phosphorylation might instead shift LARP1 binding preference to mRNA targets with different isolation and/or cross-linking efficiency.

#### Subcellular Localization

RNA-binding proteins are often localized to specific subcellular compartments where they interact with their targets (Sundararaman et al., 2016). This is important since the local concentrations of RBPs and target RNAs affect their interaction. PTMs in several RBPs have been shown to influence subcellular localization (Thapar, 2015; Lorton and Shechter, 2019). ELAVL1 (a.k.a. HuR) is a well-studied example for this: Phosphorylation of several sites near a nuclear localization signal induces protein accumulation in the cytosol, where it binds to and regulates mRNA targets stability (Abdelmohsen et al., 2007; Doller et al., 2007; Kim et al.,2008a,b; Lafarga et al., 2009). As is the case for ELAVL1, shuttling between subcellular compartments affects RBP interaction with RNA targets individually, increasing the interaction with some and decreasing with other RNAs. UV cross-linking and RNA isolation are also specific to each RBP-RNA pair. Altogether, it is very difficult to predict fold changes in RIC-like experiments following subcellular localization regulation of RBPs by PTMs.

#### Protein–Protein Interaction and Complex Formation

An important function of many RBPs is to bring target RNAs in contact with core ribonucleoprotein machineries, like the exosome, the ribosome and the spliceosome (Gehring et al., 2017). Several PTMs have been shown to regulate formation and stability of protein–protein interactions in RBPs with consequences for target RNAs (Zarnack et al., 2020). The consequence of such regulation for quantification in RIC-like experiments will depend on the protein partners. For instance, phosphorylation of NCL activates the deadenylase activity of its binding partner PARN, leading to shortening of poly-A tails in NCL-targeted RNAs (Zhang et al., 2018). Another example is phosphorylation of UPF1, which triggers formation of the RNA-decay complex and degradation of UPF1-bound RNAs (Durand et al., 2016). In both cases, phosphorylation is expected to affect pulldown efficiencies in RIC-like experiments without necessarily changing RNA-binding.

#### Phase Separation

Posttranslational modification of RBPs recently emerged as important regulators of liquid-liquid phase separation and ribonucleoprotein granule dynamics (Hofweber and Dormann, 2019). Particularly, methylation of arginine- and glycine-rich regions in RBPs plays an important role (Hofweber et al., 2018; Qamar et al., 2018). During phase separation, proteins interact with other proteins and RNAs to form membraneless condensates. RBP arginine methylation affects this condensation and thereby likely changes the set of RNAs bound by an RBP. On the one hand, it is not known if RNAs in condensates are efficiently isolated in RIC-like experiments. On the other hand, as discussed above, selection of RNA targets might lead to differential quantification in RIC-like experiments due to altered RNA isolation and UV cross-linking efficiency. Therefore, even though RBPs might interact more with RNAs in condensates, it is not clear if this results in corresponding changes in RIC-like experiments.

## Discussion

The last decade has seen great advances in the systematic identification of RBPs (Gebauer et al., 2020). In particular, CLIP- and RIC-like approaches provide global pictures of RBPs and their target RNAs. While the number of known RBPs now exceeds the number of known transcription factors, we are just beginning to understand how cell signaling and PTMs affect their function. In contrast to transcription factors that interact with an essentially constant genome, the fact that the transcriptome is highly dynamic complicates interpretation of RBP function. Here, we discussed challenges involved in interpreting CLIP and especially RIC-like results quantitatively and presented selected examples of how PTMs in RBPs could affect quantification. Particular qualities of the RBP (cellular functions, bound RNAs, protein interactors, etc.) and aspects of the conditions investigated (cell cycle state, global cellular adaptations to perturbation, discrepant transcriptomes, etc.) all affect the experimental results obtained. Therefore, an observed change (or lack thereof) in RIC-like experiments should not be interpreted to indicate altered (or constant) RNA-association of an RBP. Having said this, it is also important to point out that CLIP- and RIC-like methods are very powerful approaches for the analysis of posttranscriptional regulation. However, apparent changes in RNA-binding observed with these methods should not be regarded as an end in themselves but rather as starting points for deeper analyses. It is instructive to more generously interpret such changes as possible RBP perturbation events rather than increased or decreased binding. It is then important to take a closer look at the biology of the protein under study and consider also other factors that might affect pull-down efficiency, besides RNA-binding. These factors include (but are not limited to) the examples given above, like changes in subcellular localization, altered protein-protein interactions, global proteome and/or transcriptome changes, phase separation, and switching between the classes of RNAs bound by an RBP.

## Data Availability Statement

Data presented in this study was directly obtained from the respective references. No new data was generated for this work.

## Author Contributions

CHV-V analyzed data and prepared figures with input from MS. CHV-V and MS wrote the manuscript. Both authors contributed to the article and approved the submitted version.

## Conflict of Interest

The authors declare that the research was conducted in the absence of any commercial or financial relationships that could be construed as a potential conflict of interest.
